# A qualitative assessment of medical assistant professional aspirations and their alignment with career ladders across three institutions

**DOI:** 10.1186/s12875-022-01712-z

**Published:** 2022-05-16

**Authors:** Stacie Vilendrer, Alexis Amano, Cati Brown Johnson, Timothy Morrison, Steve Asch

**Affiliations:** 1 Division of Primary Care and Population Health, Stanford University School of Medicine, 1265 Welch Rd., Mail Code 5475, Stanford, CA 94305 USA; 2 Stanford Health Care, 300 Pasteur Drive, Palo Alto, CA 94304 USA

**Keywords:** Medical assistants, Career ladders, Health care workforce, Primary care, Team-based care

## Abstract

**Background:**

Growing demand for medical assistants (MAs) in team-based primary care has led health systems to explore career ladders based on expanded MA responsibilities as a solution to improve MA recruitment and retention. However, the practical implementation of career ladders remains a challenge for many health systems. In this study, we aim to understand MA career aspirations and their alignment with available advancement opportunities.

**Methods:**

Semi-structured focus groups were conducted August to December 2019 in primary care clinics based in three health systems in California and Utah. MA perspectives of career aspirations and their alignment with existing career ladders were discussed, recorded, and qualitatively analyzed.

**Results:**

Ten focus groups conducted with 59 participants revealed three major themes: mixed perceptions of expanded MA roles with concern over increased responsibility without commensurate increase in pay; divergent career aspirations among MAs not addressed by existing career ladders; and career ladder implementation challenges including opaque advancement requirements and lack of consistency across practice settings.

**Conclusion:**

MAs held positive perceptions of career ladders in theory, yet recommended a number of improvements to their practical implementation across three institutions including improving clarity and consistency around requirements for advancement and matching compensation to job responsibilities. The emergence of two distinct clusters of MA professional needs and desires suggests an opportunity to further optimize career ladders to provide tailored support to MAs in order to strengthen the healthcare workforce and talent pipeline.

**Supplementary Information:**

The online version contains supplementary material available at 10.1186/s12875-022-01712-z.

## Background

Primary care practices have increasingly turned to team-based primary care models in their efforts to efficiently provide high quality care [1, 2]. As work processes shift in these multi-disciplinary teams to allow each member to perform “at the top of their license” [3, 4], medical assistants (MAs) have seen their responsibilities expand to include panel management, health coaching, scribing, translating, phlebotomy, and other multi-functional roles, which vary by site and state licensing [5–7]. Demand is skyrocketing for MAs; growth projections exceed the average for all occupations by over four-fold [8]. Factors contributing to demand include the relative value of MAs in health systems (2019 median salary $34,800 [8]), short training periods, scope of work flexibility, and contribution to positive patient outcomes [5, 9].

Efforts to employ and retain such a valuable workforce are of considerable interest to healthcare organizations, given the shortage of available MAs, annual turnover rates of 20–30%, and replacement costs that reach 40% of MA yearly salary [10, 11]. Research around these challenges is limited; lack of career advancement opportunity [10, 12] and negative perceptions of organizational culture may contribute to MA turnover [13].

Given these challenges, many organizations are exploring novel solutions to recruit and retain the MA workforce [5, 14] with the goal of ultimately improving patient outcomes and workforce efficiency [15–18]. One such solution is the implementation of career ladders— paths of professional advancement that provide employees with greater compensation as they cultivate and demonstrate additional skills and increase job responsibilities [15]. Formal MA career advancement opportunities have been associated with improved quality of care, teamwork, employee satisfaction and intent to stay with current employer [5, 9, 19, 20]. An evaluation of 15 case studies in which new MA roles and opportunities for advancement were implemented alongside primary care model redesigns found associated improvements in patient and employee satisfaction, cost reduction, and quality [5]. Notably, MA advancement opportunities also have equity implications. Both women and racial minorities make up the majority of working MAs [21]. By improving wage earning and career advancement opportunities, healthcare organizations have the opportunity to address racial and gender equity in the healthcare workforce.

Despite these benefits, health organizations face challenges expanding the MA role and structuring meaningful advancement opportunities [7, 22]. Lags in implementation of a career ladder following MA role expansion can lead to MA frustration, particularly as these workers may see their responsibilities, but not pay, increase [7]. Career ladders often require institutional-level support, given that adjustments to compensation often occur at a system-wide level [7, 23]. Variation in MA training as well as in state certification and licensure requirements present further obstacles [7, 16]. MA education and training programs range from 6-month certificate programs to two-year associates degree programs, and the curriculum offered often varies between programs. Although no states require MA licensure or professional certification, many require certification in specific practice settings or require job training [16]. MA certification is offered by a number of professional and certification organizations, but the associated education and training requirements vary [19].

As healthcare organizations continue to establish and refine career ladders, an understanding of MA career aspirations and how they align with current implementations of career ladders is needed. This assessment aims to fill this gap with a qualitative analysis of MA focus groups discussing career aspirations and career ladders implemented at three institutions.

## Methods

MA perspectives of career aspirations and existing career ladders within their institutions were assessed through a series of semi-structured focus groups. Implementation outcomes were drawn from the Implementation Outcomes Framework, including acceptability, appropriateness, and perceived effectiveness at improving recruitment and retention [24].

### Settings

Sites included primary care clinics in three health systems across urban, suburban and partially rural U.S. geographies (University Healthcare Alliance, Newark, CA; Stanford Health Care, Stanford, CA; Intermountain Healthcare, Salt Lake City, UT). Within each institution, a subset of sites were chosen to represent urban (including suburban) and partial rural settings where available [25].

**Fig. 1:**
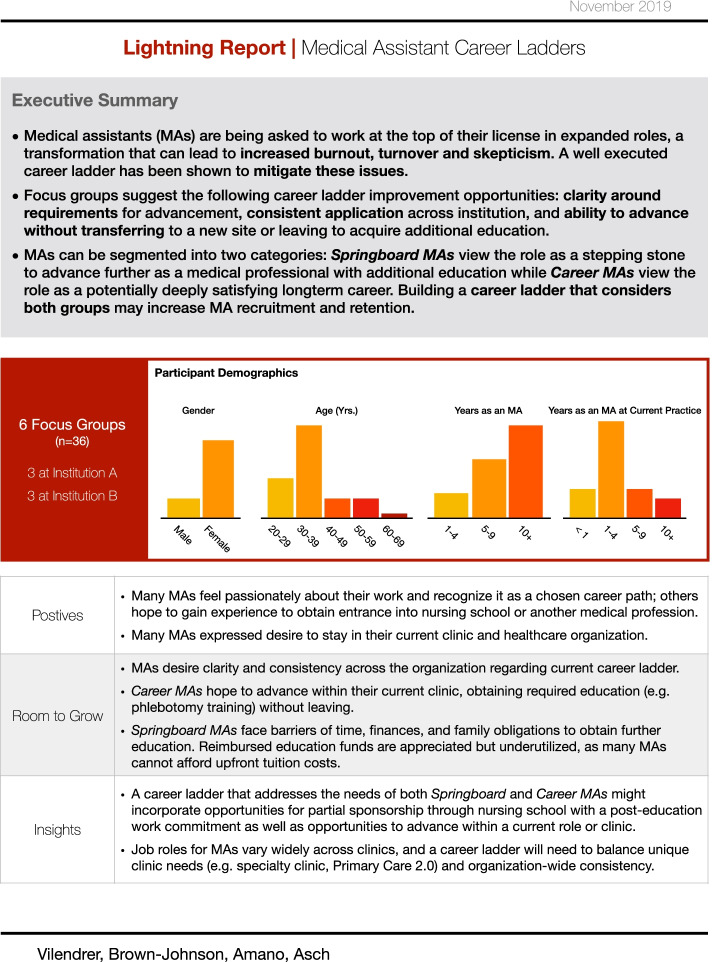
Lightning report on focus group findings

MA career ladders at the three institutions ranged between 3 and 4 levels, where combination of clinical responsibility and tenure within a site determined a promotion. Administrative contacts reported these were in place for 1 year or more across each institution, though the details varied by clinical site and were often not documented. Two of the organizations were also in the process of revising career ladder details at the time of analysis, thus it was not feasible to capture the details of each of these heterogenous career ladder structures in this analysis. This evaluation was reviewed by the Stanford School of Medicine and Intermountain Healthcare Institutional Review Boards and did not meet the definition of human subjects research; it therefore followed institutional protocols governing quality improvement efforts rather than research (Protocols #51945, #1051215, respectively). As such, early findings were reported back to operational leaders at each institution partway through the analysis to inform ongoing improvement (Fig. 1) [26]. Informed consent was obtained from all participants.

### Data collection

From August to December 2019, all MAs within each selected clinic were emailed an invitation to participate in an hour-long focus group by managers who were not present during the conversation. The focus group methodology was chosen to optimize limited research resources, draw out the collective views of the MA population and engage otherwise hesitant participants, particularly given their relative vulnerability as the lowest paid members of the clinical team [27, 28]. An unknown minority of MA participants who were invited did not attend the focus group due to clinical care activities. Participants did not receive financial compensation, though lunch was provided. No author practiced within these clinics. Focus groups (led by physician and health services researcher SV) were conducted at clinic sites and consisted of a qualitative semi-structured discussion around MA perceptions of career ladders and financial incentives, the latter of which is the focus of other work [14]. (See protocol in Additional file 1: Appendix A.) Conversations were recorded with permission from all participants and transcribed (Rev, Austin, TX). Field notes were also taken by an author (AA) in three focus groups. Data collection continued until thematic saturation was achieved.

### Data analysis

Analysis of data collected was rooted in grounded theory [29–31]. Authors (SV, CBJ, AA) created an initial codebook based on emergent themes from early transcripts and used a constant comparative method [30–32] to categorize remaining data using software (NVivo 12, Burlington, MA). Authors (SV, CBJ, AA) collectively reviewed a subset of three transcripts to reach consensus on a coding structure before recoding all remaining transcripts in sequence to ensure consistency. Codes were further analyzed by a single author (AA) to identify any potential differences in MA perceptions across clinic organizations and geographies. The consolidated criteria for reporting qualitative research (COREQ) were used to inform reporting of the study findings (Supplemental file 1) [33].

## Results

Across the three institutions, ten focus groups were conducted with 4 to 9 participants each for a total of 59 participants. Most MA participants (78.0%) worked in urban/suburban settings, 44% were 30–39 years of age, 92% identified as women, 37% were white, and 54% were non-Hispanic. Nearly half had worked as an MA for 10+ years (Additional file 1: Appendix B). The demographic composition of study participants with female sex and non-White individuals predominating was consistent with national and local trends [16, 34, 35]. Findings were consistent across institutions as well as urban versus partial rural areas and are therefore described uniformly.

Our qualitative analysis surfaced three major themes: mixed perceptions of expanded MA roles with primary concern over increased responsibility without commensurate increase in pay; divergent career aspirations among MAs not addressed by existing career ladders; and career ladder implementation challenges including opaque advancement requirements and lack of consistency across practice settings. Underlying each of these themes were feelings of underappreciation for the MA role by healthcare organizations. Beyond these themes, a full accounting of factors reported to influence MAs’ decisions to join and remain within their organization can be found in Additional file 1: Appendix C.

### Mixed perceptions of MA career ladders and expanded roles

MAs overall welcomed the existence of a career ladder that would help them understand steps to gaining skills and increasing professional and economic growth. MAs also reported expanded responsibilities at all career levels when compared with their historical roles. However, this increased responsibility was often not associated with increased compensation, which led to subthemes of burnout, frustration over licensure limitations, and skepticism about the value placed on MA’s by the organization.

Several MAs elucidated the tension of being open to more responsibilities so long as they were associated with increased compensation. One MA shared, “I think it’s [career ladder] a positive thing. Also, if they’re going to pay you more, then it’s a really, really good positive thing” (MA 1, FG 2). Another was frustrated with recent changes to her workload: “Two [years ago the work increased]…My workload’s way different …a lot more computer stuff, reports, calling patients” (MA4, FG7). MAs expressed that this felt unfair: “It’s just discouraging if we’re doing all this work, and we’re not being recognized on our title and on our paycheck” (MA8, FG6). Some expressed a desire for a return to their prior responsibilities, or reported the variety of responsibilities and sheer workload created time pressures that reduced job satisfaction.

At the same time, MAs shared frustration at the limitations of what their licenses or job responsibilities allowed them to do. This sentiment clustered around the lack of upward growth opportunities available as well as limitations in day-to-day activities. One MA expressed dissatisfaction at the loss of her ability to place intravenous lines (IVs) due to changes in institutional protocols. Activities that were valued included patient-facing interaction, minor procedures (e.g. IV placement); less valued were computer work and scheduling. Overall, MAs’ desire for increased patient-facing and procedural responsibilities was uniform and appeared conditional on having enough time during the day to complete such tasks and the recognition of this added value in their paychecks.

Underlying these sentiments was skepticism of the organizational value of MAs. This was another factor that some MAs described as driving their desire to leave a given organization. Even while MAs were reportedly in short supply, they reported hearing the message from administration that they were dispensable:

“MA1: …a lot of people say that they don't feel like they're protected here. Like you could literally get fired for the smallest things.

MA3: …I was always afraid that I was getting fired because of things that were said…And just constantly getting talked to, or at, about certain things and never having that representative for myself in there. It was always my word against the manager's word…

MA2: You feel like management is against you and trying to get rid of you kind of thing. And then when you try to reach out to HR, they kind of give you that whole, ‘It's your manager. I'm going to have his or her's back, not your back, because you're replaceable and management's not replaceable.’” (MA1, MA2, MA3, FG4)

Some MAs described their human resource contact as being largely unhelpful, particularly related to questions of work performance, promotion, or career ladders.

### Divergent MA career aspirations: “springboard” vs “career” MAs

MA career aspirations varied considerably and fell in two clusters: “springboard” MAs who pursued their current role as one step along a path to obtain a higher level license in healthcare, and “career” MAs who were not interested in obtaining a higher license in healthcare but rather were interested in growing within their careers as MAs (Table 1). Whether a given MA fell into one category or another depend in part on their backgrounds: “…personality-wise, we’re not all the same person. We have huge diversity groups in how you were raised or what your projection is or what you want out of life.” (MA 2, FG 7).

**Table 1:** Medical assistant desired career trajectories and recommended path for advancement by cluster

Cluster	Definition	Example of cluster	Direct and implied recommended paths for advancement
“Springboard” medical assistants	These individuals pursue an MA role as one step along a path to obtain a higher level license in healthcare	“I’m going to be going into nursing school, so I’m using medical assisting as a stepping stone, to get where I want to be, and also have a good resume. So, I really like it. Facilitator: Because it’s hard to get into nursing school? MA: Right. And also saving up money. (MA 4, FG 10) “I know some people want to be nurses. They’re not quite sure that they want to be an RN and be that committed. So, they try to be a medical assistant, and if that goes well, they go to the next step.”(MA 7, FG 6) “I actually want to become a primary school teacher, so this is just to save money for school. And then I’m going to go back to school… So I wanted to become an RN, but I kind of got sick of healthcare and I want to do something different.” (MA 5, FG 10)	**Offer generous post-training work agreements for loan-forgiveness** *Reported loan-forgiveness at one institution in evaluation* “MA9: There’s an agreement, where you have to stay with [health system], for a certain amount after, or you would have to pay them back…I would say three years… It’s like 1%. Interviewer: Okay, so it’s 1% of tuition cost, but in order to not pay that back, you have to work for them for three years? MA9: Right.” (MA9, FG10) **Work with local training programs to build work-study program** “…like even any leadership position or training position you have to have a BA or a BS or something like that to move up for those, so that’s where the struggle is for that, especially with the hours that we work it’s hard to do schooling to completely finish a program without leaving your job position.” (MA 2, FG 1)
“Career” medical assistants	These individuals are not interested in receiving additional training to move out of the MA role; some are interested in growing within their careers as MAs.	“MA2: I know I don’t want to be a nurse, so I’m not going to go to school for that. Would I love to make more money? Sure. Do I love my schedule here? Yes. I work four 10s, Fridays off, don’t work holidays, don’t work weekends. I don’t want to go to graveyard. I don’t want to go to weekends. I don’t want to be on call...I mean I know it’s not the best thing to do but right now I’m happy and comfortable. My bills are paid…. MA4: If you love the provider you work with, you have the perfect schedule and you’re not totally getting crappy pay then that’s a comfortable spot to be in.” (MA2, MA4, FG7) “I actually literally got into a discussion with an RN who told me that us MAs are nurse wannabes that couldn’t cut it. I was like ‘How insulting’. First off, we do this for a reason…we love the clinic setting. I said ‘you couldn’t step into my clinic and do what I did no more than I could step in and run your machines…You just couldn’t, I don’t care what your degrees.’ It’s different but the pay differences is ridiculous but what we do is equally as important as what they do. We’re not nurse wannabes. I wouldn’t be a nurse over an MA.” (MA3, FG9)	**Hire medical assistants into internal administrative roles** “I don’t know it was this year or last year, but I had heard that there was some clinic, somewhere, that [INSTITUTION] had actually hired a medical assistant as an assistant manager, and I think for a little while that was kind of motivating. Because you’re like, ‘Oh, we can actually do that?’… there’s a lot of people that are medical assistants that are actually striving to be management.” (MA3, FG4) **Offer medical assistants designated time to specialize in new responsibilities** “MA3: At my other clinic [prior to switching to current clinic] I very well would have been the managing MA that writes the schedule, covers the docs. Interviewer: So that would have been motivating for you? MA3: Oh, absolutely…Because with that came a pay raises as well.” (MA3, FG9)

“Springboard” MAs reported a desire to gain experience and save money in order to return to school primarily to become a nurse, though individuals also shared plans to become a physician or health administrator. Understanding their personal interest in healthcare before committing to additional training was felt to be a key reason for choosing the MA role: “Nursing... it’s expensive, and then it’s hard to get into. So, you don’t want to be that committed [before knowing you are ready]... I have friends that went into the medical field, and then after [they] were done or close to being done, they found out that they hate blood” (MA6, FG 6). The cost of making a mistake in investing in one’s career was thought to be high.

Alternately, “career” MAs did not nurture plans to return to school or switch professions. Instead, they expressed general contentment in their field and even described the benefits of being an MA over other healthcare careers:

“MA3:…I wouldn't even want to go to school as an RN... You just don't get that interaction with the patient…they [nurses] have time to go in, start the IV, run the machine, change bags, and then they're gone….I don't want to be that, I want to do patient interactions.

MA4: Our patients know our names.” (MA3, MA4, FG9)

A majority hoped to grow within their existing career and shared a desire to move into administration, teaching, or other leadership opportunities. Rare individuals expressed no desire to move up the career ladder. One attributed this to being late in her career:

“Maybe at age 60, I might want to retire…So, why stress myself out even further along…my mental health is something to consider too. So then, I said, ‘I'd rather leave it for somebody that's younger.’” (MA 3, FG 2)

### Career ladders fall short of meeting “springboard” and “career” MA needs

Career ladders fell short when viewed through the lens of diverse MA career aspirations. The “springboard” MAs described above who hope to return to school face challenges obtaining financial resources to pursue this education, often while balancing family responsibilities: “[Returning to school requires] debt, time. Hard especially if you have family.” (MA8, FG6) While MAs in several settings described receiving funds for continuing education for their employer, these were a small portion of what was required for additional training. One MA described a loan-forgiveness program where the health system paid a fraction of her loans in exchange for an agreement to work at the institution following training. This program did not seem to entice the MA to shift her plans.

“Career” MAs who expressed a desire to stay within their given roles and clinics still hoped for increased professional growth opportunities. Many felt this was lacking: “I’m in that mode where I’m struggling…I want to be more but I have to do X, Y, and Z, and leave where I’m currently happy at in order to do that.” (MA1, FG1) Other MAs gave clues as to what might constitute these growth opportunities within their given roles. For example, an MA reported that her friend who worked as an MA at an outside health system was eventually hired into an administrative leaderhip role without having to go back for more schooling. The MAs in this focus group agreed that such a professional growth opportunity would be motivating, though no such opportunity existed within their institution. Another participant identified that taking on a new specialized responsibility such as patient coaching might increase her job satisfaction.

### Career ladder implementation challenges across MAs

Other implementation challenges were noted across all MAs, regardless of their career aspirations. While MAs largely reacted positively to career ladders in theory, they desired increased clarity as to the requirements for advancement, consistency of these requirements across practice settings within a given institution, and local advancement opportunities.

MAs uniformly described a lack of clarity regarding career ladder details at all three health systems. The exchange below was typical across focus groups:

“Facilitator: So if you wanted to move up [the career ladder],… what would you have to do?

MA2: I have no idea.

MA4: We have no idea.” (FG3)

This challenge was attributed to a lack of communication from administration, both about the overall system and where individuals fit within that system: “We don’t know what level we’re in.” (MA1, FG 5). Other challenges included inconsistent recognition of responsibilities, inability to advance without re-applying for an open position or specializing, lack of individual career counseling, education funds that were challenging to use in practice, and desire for greater appreciation from local physicians and the health system overall. These sub-themes have been converted to direct and implied recommendations for career ladder improvement (Table 2).

**Table 2:** Direct and implied MA recommendations for improving career ladders

Recommendation	Example Quotation
Clear and transparent expectations for advancement, regularly communicated	“[We need] a better understanding of the tiers of how you become an MA 2? How you become MA 3? What are they basing that on? What skill set? You know, because a lot of us have been doing this for like 10 years plus. So what experience skills is necessary for…And it would have to be consistent from every [health system] clinic…cause it’s not [currently].” (MA 4, FG 3)
Consistent recognition of training, experience and work responsibilities, despite variation across backgrounds and clinic location	“So, I don’t think it’s fair to be categorized under the whole [health system] because we really do more than other MAs do at other locations. I think that for our location, we should be categorized separately because we do a lot more than they do at regular [clinics].” (MA9, FG6)
Ability to advance without waiting for a role opening	“MA 1: Yeah, I would have to leave this clinic in order to just to become an M.A. 2, one level up… MA2: Yeah, cause I started at [clinic] as an M.A. 2, and I came here cause it was like less of a commute for me, and the only available spot they had was an M.A. 1, and now I’m doing so much more than I was doing over there but it’s still M.A. 1. And I have the experience, well, we all have the experience of like an M.A. 3.” (MA1, MA2, FG 3)
Ability to advance while specializing in certain tasks	“Yeah, I just know of other medical companies, very close to us, that have MAs who function as MAs, and room the patients and take care of the patients, and they have other people in their buildings who do the referrals, and do the faxes, and do the paperwork. That’s what I came from. ... So it’s separate, and it’s not merged into one position. So having it separate, and not putting all the pressure and responsibility on one person, seems to be a better...Instead of so much responsibility on one person, and then we all have burnout and don’t want to come to our job… “(MA7, FG10)
Individual career counseling	“MA1: I think they should individually sit down with you and talk to you where each of you are at, individually, that way we know where to grow, and where to become a better MA. We have our ‘yearlys’ [annual review], but it has nothing to do with this [career ladder]. MA4: I don’t have a yearly. (MA1, MA4, FG5)
Direct payment for educational opportunities out of educational funds	“I want to do my certificate, but the money is a barrier. We have the $2000 [in education credit] that we all have, but instead of [the health system] paying the money towards that, they want us to pay it directly and then they’ll refund it. I think if they can pay them directly, it will make it a little bit easier for us to do the certification for MA. Or go back to school, get the online courses, go in to become and RN or whatever someone would like to be.” (MA1, FG5)
Demonstration of appreciation from health system and local physicians	“But there’s no promotion, even if you do a perfect job, you don’t have a promotion with that. Okay. No we don’t have anything, we don’t have an employee of the month, or anything like that as this office.” (MA 1, FG 2) “You know, it’s really not just about the money, but we do so much more than a lot of our other clinics…I feel that it would be awesome to have that [increased compensation] though, and title change just to show the appreciation for the medical assistants and for how much they do.” (MA 4, FG 3)

Where career ladder knowledge existed, MAs faced other obstacles to advancement, such as the need for self-funded education: “You can become MA3, …but you have to have specific certification and you have to do CME [continuing medical education]. You have to pay for that yourself.” (MA 3, FG 7) In addition, many MAs who wanted to advance up the career ladder reported having to wait until a position of that particular level opened.

Further, MAs felt the career ladder did not acknowledge responsibility differences across clinic sites within the same institution, or differences in individual years of experience and training. Several MAs reported that job responsibilities for the same career level varied between clinics. For example, some entry-level MAs are asked to do front desk, back office, and phlebotomy work while others simply obtain vitals and room patients; MAs reported these differences were not reflected in the career ladder.

Again underscoring these concerns was a sense that MAs were not appreciated for their work. MAs highlighted the need to build this recognition into career ladder and compensation structure: “I think being more appreciated is a huge thing… knowing that I’m making a difference.” (MA5, FG9) These collective challenges made it difficult for MAs to advance within their existing role and clinic.

## Discussion

Well-designed career ladders have the potential to improve job satisfaction, thereby improving recruitment and retention of health workers with downstream benefits on patient care and operational efficiency. We found positive MA perceptions of career ladders in principle, though elements of their practical implementation were reported to need improvement across three institutions. Two disinct groups of MAs emerged with regard to their professional ambitions: “springboard” MAs hoped to advance to higher paying non-MA roles while “career” MAs desired professional and financial growth opportunities within the MA profession. Reported and implied recommendations for career ladder improvement included the need for health systems to provide MAs with clear and transparent requirements to ascend career ladders; consistent recognition of training, experience, and work responsibilities across the organization as demonstrated through career ladders; the ability to advance in place or with increased specialization; career counseling; and streamlined opportunities to use educational funds. The need for transparency and consistency in career ladder implementation is consistent with prior work [7], though this evaluation further contributes to discussions around structuring opportunities for advancement including continuing education and recognition that MAs may cluster into distinct segments based on their needs and career aspirations.

MAs varied in terms of their professional ambitions, including the degree to which they hoped to grow within their existing role and whether they planned to pursue additional training to move into another profession. Designing career experiences around employee career aspirations, including “grouping employees into clusters based on their wants and needs” has been briefly explored in business literature [36], yet such programs have yet to be formally explored in healthcare. Diverse MA needs discovered here suggest opportunities to optimize career ladders from the perspective of two distinct groups: “springboard” MAs and “career” MAs.

For “springboard MAs”, these results suggest health systems may benefit from anticipating—and moreover supporting—transitions from MA to other health professions, particularly for individuals who hope to remain within a given medical system. MAs frequently reported considering nursing as the next step in their career, a profession with well-documented worker shortages and high turnover cost [37–40]. Supporting these “springboard” MAs in their desire to become fully-trained nurses or other types of healthcare professionals may be a savvy way for health systems to create talent pipelines. We heard a single example in which one health system paid a small amount of tuition for additional education in exchange for an agreement to work after training for a minimum number of years. Such agreements exist in other industries and are increasingly used with physician trainees [41]; extending an adapted program to other health professions, including MAs, deserves further exploration.

MAs’ varied levels of ambition suggest that at least some turnover should be anticipated. Further study is needed to quantify the impact MA career intentions have on turnover, including the portion of MAs who may be retained or positively directed towards other roles within a given health system. We also note that supporting such MA advancement opportunities—whether within the MA role or in non-MA roles within the same institution—may benefit institutional goals towards diversifying workforce and leadership, as MAs typically come from diverse backgrounds that often closely align with the patient population they serve [5, 34].

For the “career” MAs, we heard that opportunities that allow for advancement within their current MA profession may increase job satisfaction and thereby retention with its downstream financial and organizational benefits [10, 11]. Literature outside healthcare also suggests that organizations can benefit when promoting from within, given that employees retain institution-specific knowledge that increases productivity [42, 43]. We note major barriers to facilitating MA advancement within their current roles include licensing restrictions and common staffing structures in primary care—MA role expansion may mean MAs have taken over the historical positions they might have once stepped up into. Some primary care settings, including those in this analysis, are actively exploring further specializing MA roles based on additional training in mental health, population health management, or value-based care [5, 44]. These opportunities may facilitate higher level advancement-in-place opportunities for MAs without requiring years of additional training.

We recognize another tension in that local clinic needs can vary significantly, and each may require different competencies from their MAs (e.g. phlebotomy, population health measures). This goes against MAs’ voiced desire that a career ladder consistently reflect competencies across an organization. Based on our overall findings, is seems that allowing for some local clinic-level flexibility to facilitate advancement-in-place opportunities may outweigh MA desire for career ladder consistency across the organization. Administrators must recognize and balance this tension in their efforts to optimize career ladder design.

Underlying these conversations was the dominant theme of MA role expansion in the last several years. While prior work has largely emphasized the benefits of this transition [5, 15, 16], we were struck by the unfavorable perspectives many MAs held when role expansion was discussed in the context of their career progression and, indirectly, compensation. In particular, MAs seemed to recognize they were providing more value to the health system than before, generally without increased compensation; this manifested in the perception that their organizations did not value them. It appears that career ladders, if implemented effectively, may begin to combat negative MA perceptions of fairness in their workplace, thereby improving “organizational justice” and retention [45, 46]. Fortunately, despite these sentiments, early literature based on a subset of the population represented here suggests MAs do not experience significantly elevated rates of burnout [13], though additional study is needed.

Focus groups within three institutions across two geographies cannot encompass the full range of MA perspectives across the U.S., particularly as licensing laws vary from state to state. This evaluation reflects learnings to inform institutional practices, and extrapolation to outside settings is therefore limited. Future efforts to understand and optimize career ladders may benefit from expanded participation from administrators and MAs from diverse settings. Furthermore, we acknowledge two institutions at the time of interviews were working on career ladder improvements; this period of ongoing change may have reduced overall MA knowledge and satisfaction with the pre-existing programs. Additionally, we are unable to provide specific examples of the career ladders at each institution due to variation between clinic sites and ongoing revisions to their structures; understanding these trends is an area for future research. Our use of focus groups may also have limited certain individual disclosures, though we felt the benefits from a synergistic discussion with multiple voices outweighed that risk. Finally, the focus group structure also prohibited us from making comparisons on MA perceptions between racial/ethnic groups, which may be an important area of future study.

## Conclusions

MA roles have undergone significant expansion in recent years, and identifying the right balance between organizational and employee needs is ongoing. Career ladders are perceived favorably by MAs in principle but their practical implementation merits further attention. Segmenting MAs into distinct clusters based on their career aspirations may serve as a useful model to further tailor career ladders to employee needs, though additional evaluation is still needed. Such efforts have the potential to strengthen the healthcare workforce and talent pipeline, with downstream benefits to patient care and operational efficiency.

## Supplementary Information

**Additional file 1.**

## Data Availability

Original qualitative interview recordings and transcripts will not be shared given possible risk to individual privacy. For questions related to the data, please contact Dr. Stacie Vilendrer at staciev@stanford.edu.

## Abbreviations

MA: Medical assistant
FG: Focus group
CME: Continuing medical educator
