# Supplementary material for: A qualitative assessment of medical assistant professional aspirations and their alignment with career ladders across three institutions
Source: BMC Prim Care. 2022 May 16;23:117. doi: 10.1186/s12875-022-01712-z (PMC9109348; doi:10.1186/s12875-022-01712-z)
Supplement: Supplementary file 1 — Additional file 1. [file 12875_2022_1712_MOESM1_ESM.docx]

**SUPPLEMENTARY APPENDIX**

**Appendix A: Semi-Structured Focus Group Protocol**

***Perceptions around MA Career Ladder***

1. My understanding is that [INSTITUTION] hopes to continually improve the MA career ladder. Tell me what you know about the current MA career ladder?
   1. How are you currently promoted?
2. What do you think about the career ladder?
   1. What have you liked about the career ladder?
   2. What do you dislike about the career ladder?
   3. How would you improve it?
3. When your medical assistant colleagues leave this job, why do you think they are leaving?

**Appendix B: Demographics of Medical Assistant Focus Group Participants**

| Demographic | N | % |
| --- | --- | --- |
| Age | 14 | 23.7 |
| 18-29 |  |  |
| 30-39 | 26 | 44.1 |
| 40-49 | 11 | 18.6 |
| 50-59 | 6 | 10.2 |
| 60-69 | 2 | 3.4 |
| Gender | 1 | 1.7 |
| Missing |  |  |
| Woman | 54 | 91.5 |
| Man | 4 | 6.8 |
| Race | 11 | 18.6 |
| Missing |  |  |
| American Indian | 1 | 1.7 |
| Asian | 9 | 15.3 |
| Native Hawaiian or Pacific Islander | 1 | 1.7 |
| White | 22 | 37.3 |
| Other or Multi-race | 15 | 25.4 |
| Hispanic | 32 | 54.2 |
| No |  |  |
| Yes | 27 | 45.8 |
| Urban Rural^a^ |  |  |
| Urban | 46 | 78.0 |
| Partial Rural | 13 | 20.0 |
| Health System Organization | 16 | 27.1 |
| UHA |  |  |
| SHC | 20 | 33.9 |
| IHC | 23 | 39.0 |
| Years as MA |  |  |
| <1 yr. | 2 | 3.4 |
| 1-4 yrs. | 11 | 18.6 |
| 5-9 yrs. | 17 | 28.8 |
| 10+ yrs. | 29 | 49.2 |

^a^Participants whose clinics were in counties with >25% rural area based on U.S. Census definitions were designated as partial rural. See: United States Census Bureau: Urban and Rural. Published February 24, 2020. <https://www.census.gov/programs-surveys/geography/guidance/geo-areas/urban-rural.html>.

**Appendix C: Medical assistant considerations related to recruitment and retention**

| **Consideration** | **Definition** | **Example Quotations** |
| --- | --- | --- |
| Economic growth opportunity | Degree to which MAs are able to increase their compensation | ***Retention Context***  “I tapped out [left organization]. There was no more growth at all in regards to getting pay increases.” (MA7, FG6)  “[People leave because they are] probably burnt out or promised something that doesn't get followed through…I already know that I'm topped out based off of the last raise that we were supposed to get….” (MA1, FG5) |
| Professional growth opportunity | Degree to which MAs are able to increase their professional skills by learning new tasks and/or gaining new responsibilities | ***Retention Context***  I feel like it's more I've been in this position now for 17 years, almost 18 years as a medical assistant in different facilities, different companies. And I just learned a lot through the years, so I have a lot of knowledge and stuff. And I feel like as the older I get, if I was a nurse, I could go on and be like a manager, or a lead, or a teacher. But I don't feel like I can do that as an MA. (MA1, FG 4)  “So, a lot of my friends have moved over to [competitor health system] because of the advancement in leadership where it can lead to private management. They pay less, but they promote higher. For example, I think the starting rate is [lower than nat’l average] over there, which is discouraging, but the possibilities are more endless there than here.” (MA7, FG6) |
| Compensation that reflects job responsibilities | Degree to which pay increases as responsibilities increase | ***Recruitment context***  “One of my friends almost got hired, but she denied it. She worked at urgent care, and then she found out the things we have to do because she thought she was going to get more pay because more responsibility. [When she learned this wasn’t the case], she declined it…She said that her life was already hectic. She didn't need to make it more hectic for same pay. If it was more, then it would be worth it.” (MA6,FG6)  ***Retention context***  “MA1: I think the responsibilities and the pay, there's a huge difference because, just an example, what I get paid here now to do the amount of work that I do…At [competitor institution], I get the same amount of pay, and I do way less work.  MA3: Yeah.  MA5: They're not even doing Injections or blood draws….  MA1: So I think that really attracts a lot of people to move elsewhere.” (MA1, MA3, MA5, FG4) |
| Job security | Degree to which a worker feels the organization is looking out for their best interests and protects their job | ***Retention context***  “MA1: …a lot of people say that they don't feel like they're protected here. Like you could literally get fired for the smallest things.  MA3: …I was always afraid that I was getting fired because of things that were said…And just constantly getting talked to, or at, about certain things and never having that representative for myself in there. It was always my word against the manager's word…  MA2: You feel like management is against you and trying to get rid of you kind of thing. And then when you try to reach out to HR, they kind of give you that whole, ‘It's your manager. I'm going to have his or her's back, not your back, because you're replaceable and management's not replaceable.’” (MA1, MA2, MA3, FG4)  “I almost feel a bit villainized. If I say anything, if I complain or say, ‘Hey, maybe this’. It's almost like you're afraid of stepping on toes or walking on eggshells... Because you can get written up and then that's the risk of being fired. They want to hear our opinions. But when we do [share opinions]…it's just shut down.” (MA5, FG8) |
| Fair compensation relative to internal and external peers | Degree to which compensation is felt to reflect level of training and experience, relative between employees within an organization and relative to regional market | ***Recruitment context***  “So, right now supposedly, I'm the lead MA. So I have the title…I'm the lead MA but…my badge doesn't say it. I don't know if my pay also says it. I did [get a raise upon receiving this title]. But, it wasn't…when I looked online [at competitor organizations]…it would have been way different than what I'm getting here…More.” (MA4, FG2)  ***Retention context***  “MA2: It took me 10 months full time [to complete MA certification], which is frustrating because I paid $10,000 for my education, which is way more now. And then they're hiring people off the street that we are training.  MA3: That are making the same as you.  MA1: Or more.  MA2: Some left because they found out that the people they were training were making more money than them.  MA3: That have been here for five years…  MA1: But they don't realize that what they [administration] are doing is making the MAs that are actually certified frustrated and that's what's making them leave.” (MA1, MA2, MA3, FG8) |
| Lasting interest in the healthcare field | Degree to which the content of their work in healthcare remains interesting | ***Retention context***  “MA2:…I've thought about it over the years. I don't want to be an RN now…I won't go back to school for that...Because I'm going to leave healthcare…It's 14 years [working as an MA] in January and I'm done….  MA3: So I wanted to become an RN, but I kind of got sick of healthcare and I [also] want to do something different.” (MA2, MA3, FG10) |
| Appropriate workload | Perception that the amount of work is manageable given the alotted time to complete it | ***Retention context***  “MA2: [It is] hard to get off on time and when you have like a babysitter and a baby, like you have to pick them up. MA3: And you can't leave until the doctors are done. MA4: Yep….I'll sit down at the end of the day cause we don't have any time and there's 50 messages I have to answer.  MA3: We can't have overtime, but we can't do our job. We can't do everything we need to do in the amount of the time we have.” (MA2, MA3, MA4, FG8) |
| Social attachments to coworkers | Degree to which social relations with coworkers contributes to job satisfaction | ***Retention context***  “I've been here with [health system]… for 14 years now…and last year I felt…I couldn't take it anymore with everything they piled on us…I left last year, but the minute I walked into my new job I thought, hell, have I done? Because I've worked here so long. And even though the [original] job is stressful and hard and you go home tired... And the raise is a slap in the face each year… I love everybody here [original job]... That makes a difference in staying as well…this is my family.” (MA4, FG8) |
